# Mitochondrial Dysfunction in Down Syndrome associated Alzheimer's Disease

**DOI:** 10.1002/alz70855_106248

**Published:** 2025-12-24

**Authors:** Keith P Smith, Taylor A. Strope, Brittany M Hauger, Cynthia Shaddy‐Gouvion, Mohammad Haeri, Elizabeth Head, Heather M Wilkins

**Affiliations:** ^1^ 3901 rainbow blvd, Kansas City, KS, USA; ^2^ University of Kansas Medical Center, Kansas City, KS, USA; ^3^ University of Kansas Alzheimer's Disease Research Center, Kansas City, KS, USA; ^4^ University of Kansas Alzheimer's Disease Research Center, Fairway, KS, USA; ^5^ School of Medicine, University of California, Irvine, Irvine, CA, USA; ^6^ Alzheimer Biomarker Consortium‐Down Syndrome, Irvine, CA, USA

## Abstract

**Background:**

Mitochondrial dysfunction and Aβ accumulation are hallmarks of Alzheimer's disease (AD). However, the role of these pathologies in Down Syndrome associated Alzheimer's Disease (DSAD) is unknown. Here we interrogated mitochondrial function and proteomics, mitochondrial APP localization, and AD pathological hallmarks in DSAD.

**Method:**

Non‐age matched ND (*n* = 10, without DS or AD) and DS associated Alzheimer's Disease (DSAD, *n* = 10), and mosaic DS cases (*n* = 3) postmortem brain tissue was obtained from the University of California Irvine/ABC‐DS. Age matched ND human postmortem brain samples (*n* = 10, without DS or AD) were obtained from the NIH brain biobank. Age matched ND and sporadic AD (sAD) postmortem brain samples were obtained from the KU‐ADRC. Human iPSC lines were purchased from WiCell or obtained from the Linda Crnic Institute with Trisomy 21 and isogenic control lines which underwent Crispr/Cas9 genome editing to remove the extra chromosome 21 copy. iPSC models were differentiated into cerebral organoids using StemCell Technologies reagents and protocols. We examined mitochondrial function using a Seahorse XF analyzer. We measured full‐length APP protein levels in whole cell extracts and mitochondrial fractions via Western Blotting. We measured Aβ levels with ELISA kits from ThermoFisher. We also completed proteomics on the human postmortem whole brain and mitochondrial samples.

**Result:**

DSAD postmortem brain tissue had reduced mitochondrial function regardless of sex. Full‐length APP levels were significantly higher in mitochondrial fractions in DSAD brain tissue. Mosaic DS cases did not have improved mitochondrial function compared to DSAD cases. When compared to sAD postmortem brain samples, DSAD postmortem brain tissue has more profound mitochondrial dysfunction. Cerebral organoid models showed similar phenotypes to postmortem brain tissues, including increased mitochondrial APP levels and decreased mitochondrial function. Proteomic analysis identified inflammatory and mitochondrial proteins and pathways were differentially expressed and regulated in DSAD postmortem brain.

**Conclusion:**

We describe profound mitochondrial dysfunction in human postmortem brain from DSAD individuals which is recapitulated in cerebral organoid models. Mitochondrial fractions also show increased APP accumulation.

